# Enriching Zoo-Housed Ring-Tailed Lemurs (*Lemur catta*): Assessing the Influence of Three Types of Environmental Enrichment on Behavior

**DOI:** 10.3390/ani12202836

**Published:** 2022-10-19

**Authors:** Marta Caselli, Patrizia Messeri, Francesco Dessì-Fulgheri, Francesca Bandoli

**Affiliations:** 1 Department of Life Sciences and Systems Biology, University of Torino, Via Accademia Albertina 13, 10123 Torino, Italy; 2 Department of Evolutionary Biology, University of Firenze, Via Madonna del Piano 6, Sesto Fiorentino, 50019 Firenze, Italy; 3 Giardino Zoologico di Pistoia, Via Pieve a Celle 160a, 51100 Pistoia, Italy

**Keywords:** *L. catta*, zoo, enrichment, behavioral management, animal welfare

## Abstract

**Simple Summary:**

Environmental enrichments are frequently used in zoos and aquaria to enhance animal welfare by adding or changing enclosure features and providing animals with new stimuli. We tested three types of enrichments on a zoo-housed group of *Lemur catta* to assess the integrated effect of enrichment items and environmental and individual factors on the animals’ behavior. We collected data from June to September 2013 using the continuous animal sampling method for a total of 107 hours of observation. We observed the lemurs across five conditions (i.e., baseline, food-related, physical, auditory enrichments and no enrichment provided). We found that enrichments decreased stress-related behaviors, whereas the other behavioral patterns were mainly influenced by environmental and individual parameters. Our results confirmed the importance of applying multivariate research methods to properly evaluate enrichment programs and provided the hosting institution with key information to improve the lemurs’ husbandry and care.

**Abstract:**

Environmental enrichment is a management tool used to promote positive animal welfare by stimulating species-specific behaviors and providing animals with opportunities to exert choice and control over the environment. Our study aimed to evaluate the combined effect of three enrichment types and environmental/individual factors (i.e., individual age and rank position) on the behavior of six adult *Lemur catta* hosted at Pistoia Zoo (Italy). We collected data from June to September 2013 using a within-subject experimental design consisting of five conditions: Baseline, Food-based enrichment, Physical enrichment, Auditory enrichment and No enrichment provided. We conducted six 30-minute observation sessions per sampling day (total = 107 h). We recorded the animals’ behavior via 2-minute focal animal sampling per individual per observation period and analyzed data with Generalized Linear Models. The study group only performed normal species-specific behaviors. Enrichments decreased stress-related behavioral patterns, whreas environmental and individual factors influenced the other recorded behaviors. Our study confirmed the usefulness of employing an integrated methodological approach to enrichment assessment for enhancing captive lemur care.

## 1. Introduction

Environmental enrichment is one of the key tools employed by the community of zoos and aquaria to manage both domestic and wild animal species and promote positive animal welfare states [1,2]. Environmental enrichment can be defined as an animal husbandry technique aimed at enhancing captive care by providing animals with new stimuli to promote their psychological and physical well-being [1,2,3,4,5]. Indeed, animals in the wild are exposed to changing environmental conditions and cues, whereas captive animals usually have access to a limited array of stimuli compared to their wild counterparts [1,5,6]. For example, they may have to deal with the lack of space and enclosure complexity, predictable husbandry schedule and reduced choice and control with detrimental consequences on their welfare [7,8,9].

Environmental enrichments have been traditionally employed to increase the expression of species-specific behavioral patterns and reduce the occurrence of abnormal repetitive behaviors (i.e., repetitive, unvarying and seemingly functionless behavioral patterns) and stress-related ones which usually indicate poor welfare conditions [10,11,12,13]. They are commonly divided into five overlapping categories in relation to their main characteristics and aims (food-based enrichments, physical enrichments, sensory enrichments, social enrichments and cognitive enrichments [1]). Despite the fact that the evaluation of enrichment items should be a key part of any enrichment program [14], most enrichment studies have focused on large and charismatic species, such as ursids, felids and great apes [12].

We focused our study on the ring-tailed lemur (*Lemur catta*), a strepshirrine species commonly found in zoos [15]. *L. catta* is listed as endangered in the Red List of Threatened Species of the International Union for Conservation of Nature (IUCN) [16]. In captive settings, ring-tailed lemurs are housed as part of cooperative breeding and management programs aimed at maintaining genetically healthy populations in captivity for future restocking and reintroduction initiatives according to the One Plan Approach promoted by the IUCN [17].

Ring-tailed lemurs inhabit southern and southwestern Madagascar and are found in forested areas (e.g., gallery and spiny forests) and in outcrop forest patches [18,19]. They are a social and highly despotic species characterized by a strict female dominance [20]. *L. catta* lives in female-bonded groups containing approximately 10 to 25 individuals with males dispersing once they reach sexual maturity [21]. They have been generally considered diurnal, but studies in the wild have detected cathemeral activity in some populations [22,23]. They have adapted to live both on trees and on the ground where they can spend up to 33% of the day [24] and are opportunistic frugivore/folivore primates that occasionally feed on invertebrates and small vertebrates [25,26,27,28]. Despite the species’ conservation status and abundance in zoos, enrichment-related literature is still sparse, making *L. catta* a good model species to test various types of enrichments.

Previous studies on lemurs found a significant increase in activity (e.g., locomotion) and foraging behaviors thanks to the implementation of food-based enrichments (*Lemur catta* [15]; *Varecia variegata* [29,30]; *Eulemur macaco macaco*, *Lemur catta* [31]; *Lemur catta*, *Varecia rubra* [32]; *Eulemur collaris*, *Lemur catta* [33]).

Available research on sensory enrichments (e.g., auditory, olfactory and visual enrichments [1]) mostly focused on testing olfactory stimuli that often had a limited effect on lemur behavior and time budget or lead to conflicting results on their impact on the overall welfare of the studied animals (see for example: *Lemur catta*, [34]; *Varecia rubra* [35]). Interestingly, Laméris and co-authors [36] analyzed the integrated effect of enclosure type (indoor vs. outdoor) and enrichment provision (food-based and olfactory stimuli) on the behavior of a group of ring-tailed lemurs and found that enclosure characteristics had a greater influence in comparison with the implemented enrichments. Finally, a recent study on physical enrichments [37] found that sleep environments enriched with soft and insulated material raised the duration of sleeping bouts in four lemur species, including *L. catta*, with potential benefits for the animals’ well-being.

Captive lemurs were reported to develop abnormal repetitive behaviors (ARBs) such as locomotory pacing (i.e., moving repetitively along the same route) and head throw-back [31,32], as found in other non-human primates [38,39]. In addition, studies on *L. catta* [40,41] demonstrated that self-directed behaviors, such as self-grooming, self-scratching and yawning are good indicators of short-term distressed emotional states (e.g., anxiety) as in other non-human primates [42]. Various scholars highlighted a decrease of ARBs and stress-related behavioral patterns in captive non-human primates, including *L. catta*, thanks to the implementation of environmental enrichments, confirming their positive effect on animals’ overall welfare [12,35].

In zoo studies, environmental conditions and visitor presence need to be carefully taken into consideration to better assess any behavioral variation potentially associated with the changes applied to animal husbandry and care practices [1,43]. For example, a recent study analyzed the integrated impact of environmental factors and visitor attendance on a group of captive ring-tailed lemurs and found that environmental conditions influenced behavior more than visitor presence [44]. Additionally, when conducting research on social and despotic species, such as *L. catta* [21], and on groups with different age classes, individual rank and age are worth including in the analysis [1,3,4]. Indeed, enrichment items could be monopolized by high-ranking group members causing an increase of agonistic behaviors and/or could be mainly used by young subjects if energy consuming [45].

In this study, we test the combined effect of three types of environmental enrichments (i.e., food-based, physical and auditory) and environmental/individual factors on the behavior of a group of zoo-housed ring-tailed lemurs. The general aim of the study is to promote the expression of species-specific behavioral patterns and reduce ABRs and stress-related behaviors. In particular, thanks to the social structure and stability of the selected group [46] and the predictable husbandry routine [7], we expect to find low levels of agonistic, explorative, scent-marking, foraging and locomotor behaviors, and a higher level of resting and stress-related patterns (e.g., yawning, self-scratching and self-grooming [40,41] compared to studies performed in the wild, as reported also by previous research on captive lemurs [16,30,32,36]. We also expect to detect the occurrence of ARBs, as found by other scholars, and a higher level of affiliative behaviors with respect to other captive studies conducted on breeding groups [16,36]. We hypothesize that the selected enrichment items will increase the expression of exploratory, foraging, locomotion and scent-marking patterns, reduce resting and stress-related behaviors, and have a neutral effect on affiliative and agonistic ones.

## 2. Materials and Methods

### 2.1. Subjects and Study Sites

The study was conducted on a family group of 6 adult ring-tailed lemurs (mother with four adult female offspring and one adult castrated male offspring) housed at Giardino Zoologico di Pistoia (Pistoia, Italy). All the lemurs were born in captivity and were mother-reared. A list of the subjects is reported in Table 1. They were hosted in a naturalistic exhibit consisting of an outdoor section of 100 m^2^ connected to an indoor facility of 15 m^2^ composed of two rooms. Visitors could observe the animals through a glass panel located along the eastern side of the outdoor enclosure. The lemurs had 24-hour access to the indoor facility where they were fed twice a day (i.e., morning and afternoon) with mixed fruits and vegetables. The animals were managed in free contact [1] with interaction with zookeepers taking place only during husbandry procedures (i.e., enclosure cleaning and food provision).

### 2.2. Enrichment Types

**Figure 1:**
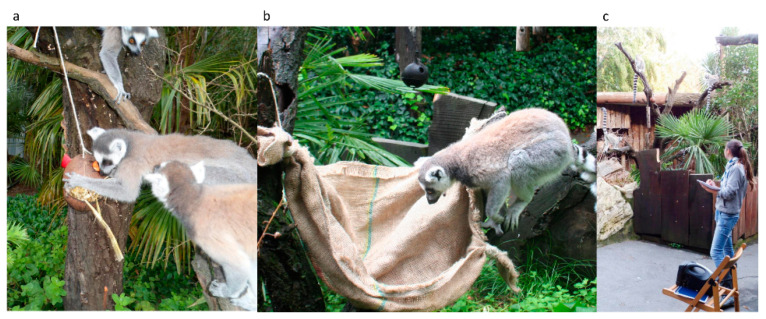
Enrichment types provided to the study group of ring-tailed lemurs: (**a**) food-based enrichment, E1; (**b**) physical enrichment, E2; (**c**) auditory enrichment, E3.

We provided the lemurs with three types of enrichments: (E1) food-based; (E2) physical; and (E3) auditory. E1 consisted of two coconut shells (diameter = 10 cm) tied together and hung on a branch with a rope (Figure 1a). We offered six coconuts—one for each subject—containing one-third of the daily diet. Each shell had two 4 cm diameter holes to allow the animals to reach the food inside using their hands. As physical enrichment (E2), we used six hammocks (24 cm × 60 cm) made from burlap sacks and hung on branches and climbing structures with a rope (Figure 1b).

For the third enrichment type (E3), we used playback of territorial calls of siamangs (*Symphalangus syndactylus*) and howler monkeys (*Alouatta* spp) played from the digital archive owned by the hosting institution.

All the enrichments were novel to the lemurs. E1 and E2 were placed in the outdoor enclosure at different locations immediately before the beginning of the first observation session. Playback (E3) was played using a portable Mipron MA-101 amplifier placed in the central part of the outdoor enclosure (Figure 1c) at the beginning of each observation session.

### 2.3. Data Collection

Data collection was preceded by a 3-week training phase, during which we conducted preliminary observations with the ad libitum sampling method [47] to design an ethogram specific to the study integrating the existing literature [46]. The ethogram included 48 behaviors grouped into 12 behavioral categories (Table 2). Individual identification was based on external features, such as tail shape and muzzle color.

Data was collected during real-time observation by the same observer over a 3-month period (11 weeks) from June to September 2013 using a within-subject experimental design [47] consisting of 5 conditions: Baseline (BL); Food-based enrichment (E1); Physical enrichment (E2); Auditory enrichment (E3); and No enrichment provided (NE). Each condition consisted of seven sampling days with six 30-min observation sessions conducted from 08:30 am to 05:30 pm for a total of 107 h of observation.

BL preceded the other conditions and took place during the first two weeks of data collection. Then, we presented two types of enrichments in six out of nine weeks and all the enrichment types in the other three weeks. We provided the enrichments following a random schedule. The same enrichment type was presented with a time interval of one or two weeks between subsequent administrations. Regarding the NE condition, we collected data one or two days after the presentation of each enrichment.

We recorded the animals’ behavior via 2-minute focal animal sampling [48] per subject per observation session. The individuals were selected as focal subjects according to a random sequence. Ambient temperature was also measured at the beginning of each observation session using a Reptiles Planet^®^ digital thermometer to calculate the daily average temperature.

### 2.4. Data Analysis

For each observation session we counted the number of behavioral samples performed by each subject and assigned the total number of behavioral patterns to the corresponding behavioral category per individual per session (Table 2).

We determined the dominance relationship using the Normalized David’s Scores (NDS) [49]. To assess the individual rank, we entered the number of decided agonistic encounters per dyad into an aggression sociomatrix using the R ‘steepness’ package (https://CRAN.R-project.org/package=steepness (accessed on 13 June 2022)). We calculated individual NDSs employing a dyadic dominance index (Dij) in which the observed proportion of wins (Pij) is corrected for the chance occurrence of the observed outcome. The chance occurrence of the observed outcome is calculated based on a binomial distribution with each subject having an equal chance of losing or winning in every agonistic interaction [49]. Correction is required when the number of interactions differ between dyads. The NDSs of the study subjects are reported in Table 1.

To test the effect of the provided enrichments on behavioral categories, we performed multivariate statistical analyses on seven behavioral categories (i.e., *affiliative*, *agonistic*, *exploration and scent-marking*, *foraging*, *locomotion*, *resting*, and *stress-related*; Table 2). We ran three GLMs including *locomotion* (GLM_1_, N_focal_observation_ = 210), *resting* (GLM_2_, N_focal_observation_ = 210) and *stress-related* behavioral patterns (GLM_3_, N_focal_observation_ = 210) as dependent variables. We coded age (numeric), individual NDS (numeric), condition (factorial: BL, E1, E2, E3, NE), day of the week (factorial: weekdays, weekend days), ambient temperature (numeric), the interaction between condition and age and between condition and NDS as fixed factors. The GLMs were performed using the R-function *glm* (family = *poisson*) of the R-package *glmmTMB*.

The other selected behavioral categories (*affiliative*, *agonistic*, *exploration and scent-markin*g, *foraging*) had a distribution with a percentage of zero-valued observations ranging from 25.7% to 81.9%. Based on Desmarais and Jeffrey [50], we ran the Vuong test with the AIC- and BIC-corrections (R-function: *vuong*) to compare the Zero-Inflated Poisson Regression (ZIPR) model with the ordinary Poisson regression model and to select the most appropriate procedure. Based on the obtained results, we performed GLMs also on the other behavioral categories (*affiliative*, GLM_4_, N_focal_observation_ = 210; *agonistic*, GLM_5_, N_focal_observation_ = 210; *explorative and scent marking*, GLM_6_, *N_focal_observation_ =* 210; *foraging*, GLM*_7_*, N_focal_observation_ = 210). For GLM_5_ (target variable: *agonistic*) we could not include the interactions (condition × day of the week, condition × age, condition × NDS) as fixed factors because the predicted probabilities of one or more observations in our data frame for this behavioral category were indistinguishable from 0 or 1.

For the GLMs, we used a likelihood ratio test [51] to detect if there was a statistically significance difference between the full and the null models (ANOVA with argument *Chisq*). We applied the Nagelkerke’s psuedo R squared test to the results of GLMs to evaluate the goodness of fit of the models. Nagelkerke’s psuedo R squared can be used to assess the predictive power of the model. If the Nagelkerke’s psuedo R squared test’s value (range= 0–1) is equal to 1, then the model explains 100% of the variation in the dependent variable [52].

We then calculated the *p* values for the individual predictors using the R-function *drop1* that implements backward elimination using a *dr object* [53]. In particular, the function computes either marginal coordinate tests (if d = NULL) or conditional marginal coordinate tests (if d is positive) and drops the predictors not reported in the *scope* (i.e., a one-sided formula specifying predictors that will never be removed), returning *p* values. The result of this analysis is an object created from the original object with the predictor with the largest *p* value removed [54].

In case of significant factorial predictors, we used the Tukey test (R-package; *multcomp*) to perform all pairwise comparisons [55]. The level of probability of tests for pairwise comparisons was adjusted based on the Bonferroni correction [47]. When we found a significance of the interactions (condition × age, condition × NDS) we considered only the effect of the interaction and not the effect of the single fixed factors. Confidence intervals were calculated with the R-function *confint.lm* (R-package: *MASS).* All the analyses were performed in R version 4.2.1 [56].

## 3. Results

### 3.1. Time Budget

In BL, *resting* was the most frequent behavioral category (33.33%), followed by *locomotion* (22.40%), *stress-related* (10.67%), *affiliative* (8.29%), *foraging* (7.50%), *exploration and scent-marking* (5.38%), *out of sight* (4.85%), *vigilance* (3.88%), *self-maintenance* (2.91), *agonisti*c (0.44%) and *interspecific interaction* (0.33%). We did not detect any abnormal repetitive behavior.

### 3.2. Generalized Linear Models (GLMs)

**Figure 2:**
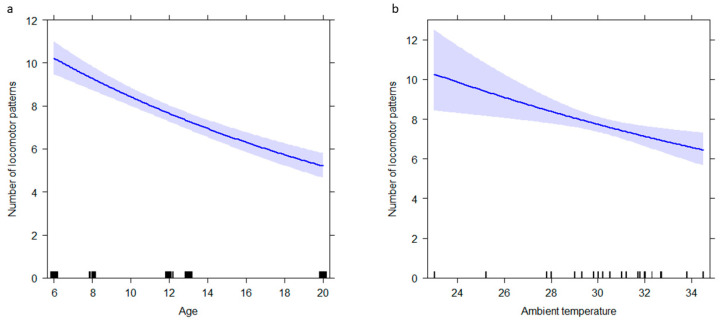
Effect plot of variables having a significant influence on the number of locomotor patterns. The number of locomotor patterns (Y axis): (**a**) decreases as the age of individuals (X axis) increases, (**b**) decreases as the ambient temperature (X axis) increases. The band represents the confidence interval.

The full model (GLM_1_; target variable: *locomotion*) including all fixed factors (age, NDS, condition, day of the week, ambient temperature, condition × age, and condition × NDS) was found to significantly differ from the null model (likelihood ratio test: *χ*2 = 127.79; *df* = 16, *p* < 0.001; Nagelkerke’s pseudo R squared = 0.46). Age and ambient temperature had a significant effect on the target behavioral category (age: *p* = 0.001; ambient temperature: *p* = 0.003; Table 3). Younger individuals engaged more in locomotion in comparison with the older ones (Figure 2a). Concurrently, the level of locomotion increased with lower temperatures (Figure 2b).

**Figure 3:**
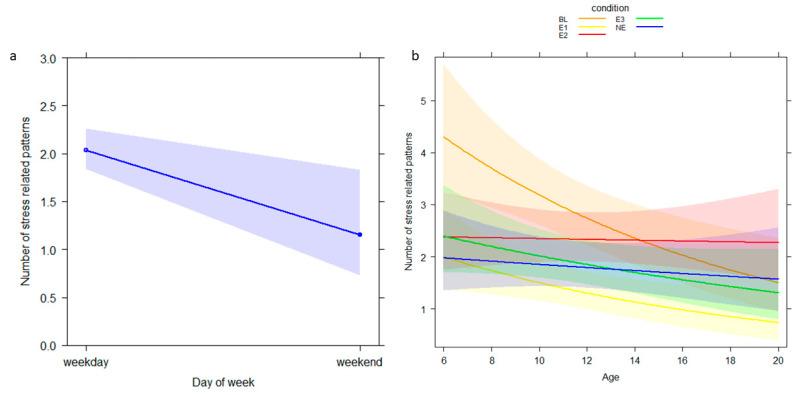
Effect plot of variables having a significant influence on the number of stress related patterns. The number of stress related patterns (Y axis): (**a**) decreases during weekend days (X axis) and (**b**) varies according to age (X axis) and condition. The band represents the confidence interval.

For GLM_2_ (target variable: *resting*), the full model did not differ from the null model (likelihood ratio test: *χ*^2^ = 13.057, *df* = 16, *p* = 0.669; Nagelkerke’s pseudo R squared = 0.06). The full model for *stress-related* patterns (GLM_3_) significantly varied from the null model (likelihood ratio test: *χ*2 = 64.328, *df* = 16, *p* < 0.001; Nagelkerke’s pseudo R squared = 0.27). We found that day of the week and the interaction between condition and age significantly predicted the level of the target variable (day of the week: *p* = 0.020; condition × age: *p* = 0.017; Table 3). The level of *stress-related* behaviors was higher on weekdays than weekend days (Figure 3a). The behavioral pattern decreased with age in all the enrichment conditions (Figure 3b).

**Figure 4:**
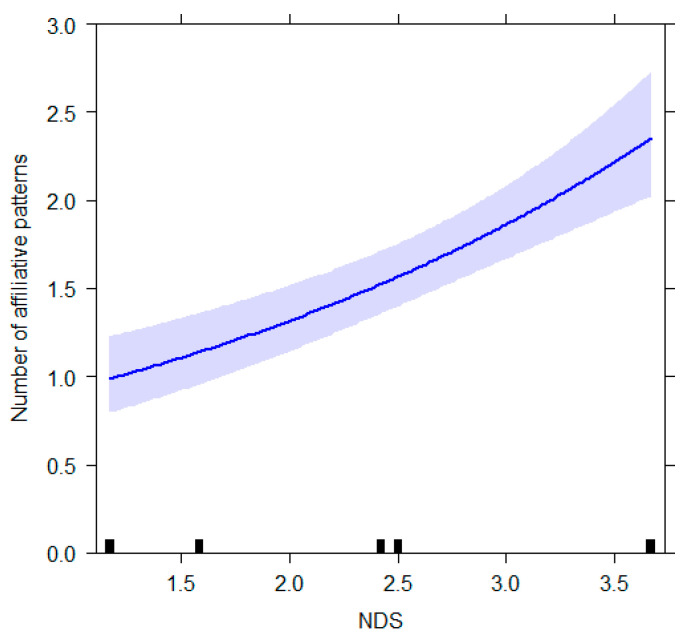
Effect plot of the variable having a significant influence on the number of affiliative patterns. The number of affiliative patterns (Y axis): increases as the NDS value (X axis) increases. The band represents the confidence interval.

The full model related to the *affiliative* behavioral category (GLM_4_) was different from the null model (likelihood ratio test: *χ*^2^ = 72.576, *df* = 16, *p* < 0.001; Nagelkerke’s pseudo R squared = 0.30). We found a significant effect of NDS on the target variable with high-ranking individuals showing the highest level of the behavioral pattern (NDS: *p* = 0.002; Table 3; Figure 4). We also detected a trend of significance for ambient temperature (*p* = 0.055) with an increase of affiliative behaviors with higher temperatures.

**Figure 5:**
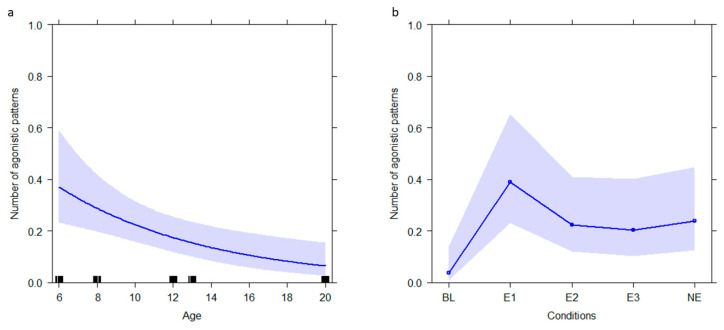
Effect plot of variables having a significant influence on the number of agonistic patterns. The number of agonistic patterns (Y axis): (**a**) decreases as the age of individuals (X axis) increases, (**b**) varies according to the enrichment condition (X axis). The band represents the confidence interval.

For GLM_5_ (target variable: *agonistic*), the full model differed from the null one (likelihood ratio test: χ^2^ = −30.414, *df* = 8, *p* < 0.001; Nagelkerke’s pseudo R squared = 0.18). Age and conditions showed a significant effect on the *agonistic* behavioral category (Table 3). Younger individuals performed more agonistic patterns than their older group mates (*p* = 0.002; Figure 5a). Moreover, agonistic behaviors were higher in E1 than in BL (Table 4; Figure 5b).

**Figure 6:**
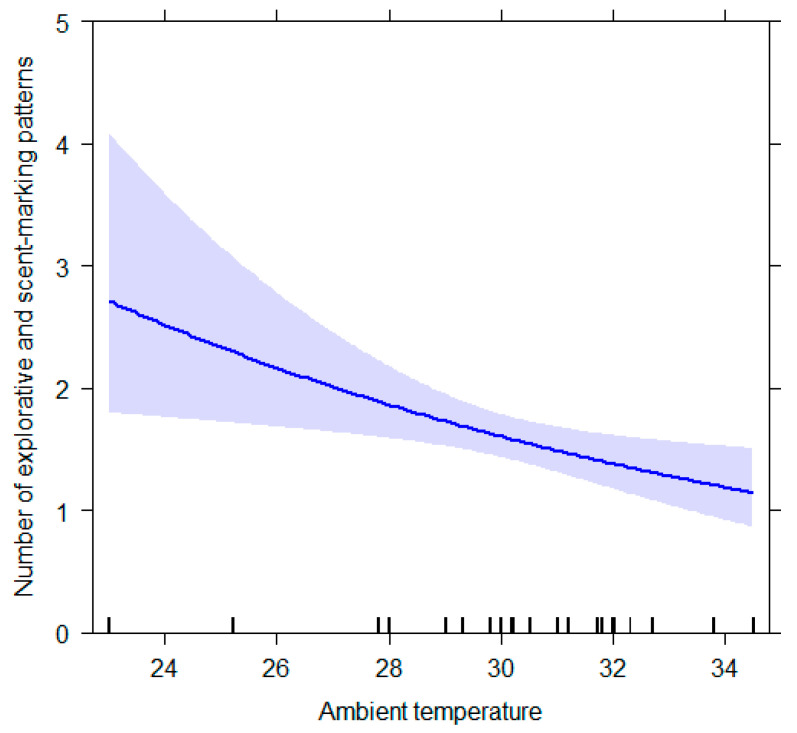
Effect plot of the variable having a significant influence on the number of explorative and scent-marking patterns. The number of explorative and scent-marking patterns (Y axis) decreases as the ambient temperature (X axis) increases. The band represents the confidence interval.

The full model for *explorative and scent-marking* behavioral patterns (GLM_6_) was found to be different from the null model (likelihood ratio test: *χ*^2^ = 41.522, *df* = 16, *p* < 0.001; Nagelkerke’s pseudo R squared = 0.18). Ambient temperature was found to be a significant predictor of the target variable (ambient temperature, *p* = 0.009). (Table 3; Figure 6). The analysis showed a trend of significance for the interaction between condition and NDS (*p* = 0.072).

**Figure 7:**
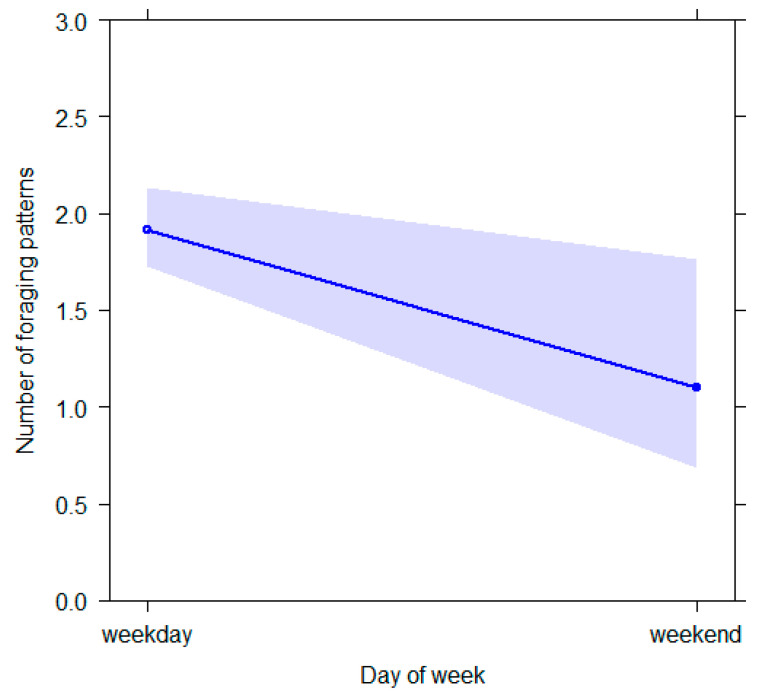
Effect plot of the variable having a significant influence on the number of foraging patterns. The number of foraging patterns (Y axis) decreases during the weekend days (X axis). The band represents the confidence interval.

For GLM_7_ (target variable = *foraging*), the full model varied from the null model (likelihood ratio test: *χ*^2^ = 29.425, *df* = 16, *p* = 0.021; Nagelkerke’s pseudo R squared = 0.13). Day of the week had a significant influence on the target behavioral pattern (Table 3; *p* = 0.025), which was performed more during weekdays. (Figure 7).

## 4. Discussion

In this section, we discuss our results regarding time budget and enrichment effect, focusing on the behavioral categories of interest (*resting*, *locomotion*, *explorative and scent-marking*, *foraging*, *stress-related*, *affiliative*, *agonistic* and *abnormal repetitive behaviors*). We only recorded normal species-specific behaviors and found a significant effect of age (*agonistic, locomotion*), NDS (*affiliative*), condition (*agonistic*), day of the week (*stress-related* and *foraging*), interaction between condition and age *(stress-related*), and ambient temperature *(locomotion* and *explorative and scent-marking*). We will also provide suggestions to improve the implementation of the proposed enrichments and discuss the importance of employing multivariate research methods to properly assess the complex range of environmental and social stimuli of captive environments.

### 4.1. Time Budget

To our knowledge, captive and wild research projects focusing on time budget [21,24,36,57,58,59,60,61] are still limited, underlying the importance of further investigating this aspect in both contexts to better compare the welfare state of captive animals with their wild conspecifics.

In our study, the predominant behavioral category of the lemurs’ time budget in BL was *resting* in accordance with data obtained both in the wild and in zoos [15,24,31,36,57,58]. Nevertheless, in contrast with our hypotheses, the subjects were found to dedicate less time to *resting* and engage more in *locomotion* compared to previous studies carried out in captivity [15,32,36,62], with an overall activity level similar to those reported for wild individuals [24,57,58,59]. Our results seem to suggest that the environment the subjects were provided with and the stimuli within and around their enclosure were effective in promoting active behaviors with potential positive welfare outcomes. Indeed, environments that do not meet species-specific requirements may result in stressful living conditions that negatively affect animals’ physical and mental health causing, for example, excessive inactivity, hyper-aggressive behaviors, and stereotypies [63,64]. Our findings are also supported by the comparable percentage of *explorative and scent-marking* behavioral patterns between our study group and wild ones [65].

In their natural habitat, ring-tailed lemurs usually spend 13–19% of their time budget moving among preferred feeding sites [59] and 18–30% foraging [21]. As expected, the study subjects engaged less in *foraging* that their wild conspecifics as found in previous research conducted in captive settings [15,36] underlying a generalized lack of species-specific foraging opportunities in zoos. Presenting food in bowls is one of the most common practices found in zoological institutions because it facilitates daily husbandry procedures and allows the animal care personnel to save working time [1]. Thus, our results, in accordance with the available literature [1], highlight the necessity to increase the complexity of food presentation in captivity to increase the time allocated to foraging behavioral patterns.

Yawning, self-scratching and self-grooming have been recognized as potential proxy of anxiety in *L. catta* [20,40,41]. For this reason, they need to be carefully considered when conducting animal welfare assessments. The percentage of stress-related behaviors in this study (10.67%) was higher than the one reported by Laméris and colleagues (5.28%) [36], potentially highlighting welfare issues. Nevertheless, differently from Laméris et al. [36] who analyzed only self-scratching and self-grooming, we included yawning in our analysis according to Zannella et al. [41]. However, the percentage of yawning in our study was 0.53%, meaning that our study group expressed more stress-related patterns in comparison to the work conducted by Laméris et al. [36].

Agonistic behaviors in the wild mostly occur around food and water resources and during mating season and intergroup encounters [66]. The study subjects were a mother with her adult offspring and the social structure of the group was stable during the observation period. Moreover, no other lemur species were housed nearby the ring-tailed lemurs and their enclosure only boarded on the north-western side with the exhibit of a colony of Greater flamingos *(Phoenicopterus roseus*) with plenty of vegetation serving as a visual barrier. As expected, we rarely detected agonistic behaviors in accordance with the available literature [36,67]. Furthermore, the expression of affiliative behaviors was comparable with the data reported on wild populations [61,65] and higher in comparison with other captive groups [15,36]. Since our study group was composed of one matrilineal lineage, this result can be explained based on the study of Taylor and Sussman [68] that reported a higher percentage of affiliative interactions between closely related individuals and matrilineal lineages.

Captive animals can develop abnormal repetitive behaviors (ABRs) that can originate from proximate and past exposure to chronic stressful stimuli. For example, ABRs could be related to limited early social experience (e.g., reduced or lack of maternal care), unsuitable environments and repeated stressful husbandry and care procedures [1,11,39]. In addition, sex, species and animal temperament was also found to potentially affect the expression of ABRs [39]. As regards non-human primates, previous studies reported various types of ABRs, such as locomotor pacing, over-grooming and over-aggression, which are usually considered a sign of potential suffering [11,38]. In our study, we only recorded normal species-specific behaviors and we did not detect any ABR, suggesting that the study subjects were provided with good housing and husbandry conditions.

### 4.2. Environmental Enrichment Effect

We found that the level of locomotion was not affected by the provision of enrichments, in contrast with previous studies on *L. catta* and other lemur species [15,29,31,32,33,36,69]. However, as reported in Section 4.1, the study group showed a higher level of locomotion compared to other research conducted in captivity [15,32,36,62] and an overall activity level comparable with wild lemur populations [24,57,58,59]. Therefore, our results underline the importance of carefully selecting the types of enrichments to induce only the needed behavioral changes. Older individuals moved less than younger ones and locomotion decreased with higher ambient temperature as reported by other scholars [32,44]

Enrichment did not have an impact on resting, in contrast with the results reported by the above-mentioned research [15,29,31,32,33,36,69] where enrichment provision was usually associated with a reduction of inactivity. However, it is worth nothing that the subjects presented a level of resting which closely mirrored those of wild conspecifics and, therefore, there was no need to further increase their activity.

The expression of stress-related behaviors was higher on weekdays than weekend days. This result could indicate a potential positive effect of visitor presence because people visited the hosting institution mainly during weekends. According to the available literature [43], visitors can exert a neutral, negative or positive effect in relation to the species, individual temperament and environmental conditions. Further studies including the recording of visitor numbers and the associated noise levels are suggested to clarify the visitor effect on stress-related patterns. The other variable that significantly affected the number of stress-related behaviors was the interaction between condition and age. Our results showed a general decrease of the target behavior with all the enrichment types with differences at individual level, thus confirming the positive influence of enrichments on animal well-being.

Interestingly, auditory enrichments are not commonly used in zoos. In a survey on enrichment practices carried out by Hoy et al. [70] and involving 60 zoos in 13 countries, 74% of respondents did not use auditory stimuli for captive mammals, despite promising results described in the literature (see for example [71,72,73,74,75]). Moreover, previous studies traditionally employed music or natural sounds (e.g., rainforest sounds) or vocalizations from conspecifics or predators [45,71,72,73,74,75]. To our knowledge, this is the first study using playbacks from primate species unknown to the target individuals. Based on our results, this type of stimulus seemed to be useful to reduce stress-related patterns as highlighted by other studies on various non-human primate species held in captivity [45,73,76,77].

Regarding agonistic behaviors, younger individual showed a higher level of agonistic patterns than older ones. Moreover, the subjects engaged more in agonistic interactions when provided with the food-related enrichment compared to the baseline, as described in previous studies on a wide range of non-human primates [45]. These results could reflect an increase of competition for the new resources available. However, it is worth noting that, despite the increase recorded with the food-based enrichment, the percentage of agonistic patterns remained low (1.55%). This result could be explained in the light of the group’s social structure that was already stable before the beginning of this study. However, it could be useful to increase the number of enrichment items and distribute them in all the sections of the enclosure to provide the subjects with multiple opportunities, as suggested in the literature [45]. 

As for affiliative patterns, we found that their expression was greater for high-ranking individuals. Although we did not distinguish between performed and received affiliative acts, we can cautiously affirm that our results are in accordance with data on affiliative interactions gathered in the wild. For example, Nakamichi and Koyama [78] reported that, in two free-ranging troops in the Berenty Reserve (Madagascar), subordinates were more likely to groom dominants than vice versa. 

Ambient temperature was found to be a significant predictor for explorative and scent-marking behaviors. As found for locomotion, increasing temperature negatively affected the number of patterns performed by the study group. According to Laméris et al. [36], it would be interesting to compare the level of exploration and scent-marking between the outdoor and indoor enclosure to verify if the indoor facility represents a less stimulating environment. Moreover, future studies could try to also implement olfactory enrichments, such as herbs, spices and essential oils, to test if this type of sensory stimuli encourage exploration and scent-marking.

Regarding *foraging*, the study lemurs foraged more on weekdays and our analysis did not reveal any significant effect of the other variables. This finding could be related to the presence of more visitors around the lemurs’ enclosure and/or higher noise levels during weekends, but additional investigations are needed to clarify this aspect. Previous studies found an increase of foraging behavior thanks to the implementation of food-related enrichments in different lemur species. For example, white-fronted lemurs (*Eulumer fulvus albiforns*) spent more time foraging when they had to retrieve food from self-operated boxes which needed manipulation to be opened [69], and the same effect was obtained for black-and-white ruffed lemurs (*Varecia variegat*a) by scattering food on their cage roof [29] and presenting fruit in trees [30]. Various authors reported a similar effect for ring-tailed lemurs with food-related enrichments ranging from scattered food to feeding devices requiring active manipulation, such as bottle feeders and tube swings [15,31,32,33]. Interestingly, research conducted by Keith-Lucas and co-authors [59] on 14 ring-tailed lemurs living in free-ranging environment found a significant increase in foraging for novel plant items. Fernandez and Timberlake [33] assessed the preference of four lemur species for different food and examined how high- and low-preferred items placed in bamboo dispensers affected behavior and enclosure use. Their results showed that high-preferred items had a greater overall effect, highlighting the importance of incorporating individual preference in enrichment planning. According to these studies, to encourage the expression of foraging behavioral patterns in the study group, the hosting institution could add novel and edible vegetation to the enclosure, as well as test food preference and use preferred items to encourage enrichment utilization.

## 5. Conclusions

Environmental enrichments are widely used by zoological institutions and need to be carefully evaluated to ensure that animals are provided with appropriate stimuli that elicit positive welfare outcomes. Our study confirmed the effectiveness of environmental enrichment in reducing the occurrence of stress-related behavioral patterns. The lack of variation in foraging behavior further supports the importance of assessing the provided stimuli. Furthermore, it highlights the necessity to take into consideration enclosure characteristics and individual preferences for specific food items when planning an enrichment program. In conclusion, our findings helped to increase the scientific knowledge of captive lemur welfare providing the animal care staff with useful information to guide practical management decisions. In addition, our study highlighted the importance of applying multivariate research methods to properly assess the impact of environmental enrichments reducing the risk of overestimating their effect. Future studies aimed at assessing the visitor effect, the time budget over the 24-hour period and the influence of enrichments implemented in the indoor enclosure are recommended to reach a more comprehensive understanding of the welfare of zoo-housed ring-tailed lemurs.

## Figures and Tables

**Table 1:** Ring-tailed lemurs involved in the study. The table reports the names of the six subjects, the year of birth, the sex and the Normalized David’s Score (used to assess the individual rank) of each subject.

Subject	Year of Birth	Sex	NDS
Milly	1993	Female	3.667
Bekili	2000	Female	2.500
Andribe	2000	Female	2.417
Mandrare	2001	Female	1.167
Ankarana	2005	Male	1.586
Sakalava	2006	Female	3.667

**Table 2:** Description of the behaviors considered for the present study based on the ethogram of Pereira and Kappeler [46], Maloney et al. [31] and Shapiro [32].

**Category 1: Locomotion**	**Description**
Locomotion	An individual ambulates on the ground or on a tree.
**Category 2: Resting**	Description
Resting	An individual remains inactive
**Category 3: Foraging**	Description
Foraging	An individual searches for and eats food with nose over the ground or terminal branch parts.
Feeding	An individual eats food provided by keepers.
**Category 4: Stress-related behaviors**	Description
Self-grooming	An individual cleans its own fur.
Scratching	An individual repeatedly and rapidly moves its hind limb digits over its own fur.
Yawning	An individual stretches mouth wide open without vocalizing in non-feeding, non-resting social context.
**Category 5: Affiliative behaviors**	Description
Sit in contact	Two or more individuals are sitting in reciprocal contact.
Proximity	Two individuals are far apart for a length not exceeding that of an arm.
Grooming	An individual cleans another one’s fur with dental comb and/or tongue. This behavior could be made or received.
Reciprocal grooming	Two individuals grooming each other.
Play	Two or more individuals play together. The most common forms of social play are the “rough and tumble”, which can include, for example, play slaps, play bites etc., and the “play run”, where one subject chases another one.
**Category 6: Agonistic behaviors**	Description
Bite	An individual bites another one.
Charge	An individual sprints < 5 m toward partner.
Cuff	An individual strikes partner (or attempt).
Full cuff	Same as cuff but some fur is pulled out.
Chase	An individual chases another one.
Muzzle	An individual has physical contact with the antagonist, but still not serious. it is used in minor situations. The animal gives a slap to the other with a quick swipe of the muzzle.
Stare	An individual widens eyes during mutual gazing with partner.
Go towards	An individual walks in a straight line towards the subject involved in the conflict. Often it assumes a gait with almost exaggerated and bold movements, moves with straight and rigid legs, keeps its head and tail very high and straight and continues to stare at the subject towards which it is heading.
Jump fight	Two individuals face each other on the ground in a race of jumping on two legs, holding their arms up and out, jumping around each other and trying to scratch, hit and bite the opponent.
Dismiss	An individual causes a lower-ranking subject to move from a certain point by a short vocalization.
Avoid	When an individual avoids interacting with another one, or when it changes its moving direction or goes far away from the latter.
Check scared	An individual who is being pursued stops and turns to look at his pursuer. The subject is in an alert position with his ears straight forward and with his eyes wide open and fixed on his pursuer.
Jump away	An individual leaps away from nearby partner (2 m)
Run away	An individual who is being chased runs away by running or jumping on branches trying to get as far away from his pursuer as possible.
Be displaced	An individual walks away after gazing at approaching conspecific.
Glance	An individual gazes rapidly toward and away from the partner.
**Category 7: Explorative and scent-marking behaviors**	Description
Skin lick	An individual licks a partner’s palms, soles, eyelids or nostrils.
Wrist mark	An individual scars arboreal substrate with carpal spur near antebrachial gland.
Wrist to pit	An individual rubs antebrachial gland against axillary gland.
Genital mark	An individual holds/rubs genitalia against arboreal substrate.
Urine mark	An individual urinates on the substrate with the hindquarters down and the tail raised like a question mark.
Anoint tail	An individual passes the ventral surface of the wrists and arms along the tail held between the legs and held erect in front of the back.
Wave tail	An individual arches his tail over his head and waves it in the direction of a conspecific to expand the smell, usually made after the anoint tail.
Sniff body	An individual places the nose less than 3 cm from the partner’s body, excluding the nose and the genital area.
Sniff genital	An individual places his nose less than 3 cm from the genital area and licks it.
**Category 8: Self-maintenance**	Description
Sun bathing	An individual sits upright in an area with sunlight, with the belly-side directed towards the sun and arms open.
Urinate	An individual eliminates urine without a specific posture but with a lowered tail.
Defecate	An individual eliminates faces without a specific posture.
**Category 9: Abnormal repetitive behaviors**	Description
Locomotory pacing	An individual walks/runs repeatedly along the same route.
Head trough-back	An individual repeatedly tosses its head in a circular motion.
Overgroom	An individual cleans itself or another individual excessively, resulting in bald patches of fur.
Self-injurious	An individual uses teeth, claws or nails to harm to itself.
**Category 10: Vigilance**	Description
Vigilance	An individual observes the surrounding environment while is sitting or standing.
**Category 11: Inter-specific interactions**	Description
Human–lemur interaction	An individual pays attention to, approaches, moves away from, etc., visitors, keepers or the observer.
Animal–lemur interaction	An individual pays attention to, approaches, moves away from, etc., other animals, such as peacocks and dogs.
**Category 12: Out of sight**	Description
Out of sight	An individual is not visible to the observer.

**Table 3:** Full results of GLMs on the effects of enrichments and environmental/individual factors on the lemurs’ behaviors (N_cases_ = 210). Significant results are reported in bold.

Predictors	Estimates	SEM	C.I.	*ꭓ^2^*	*p*
GLMM_1_ (locomotion)					
Intercept ^a^	3.476	0.450	2.589, 4.363	a	a
Age	−0.048	0.015	−0.077, −0.019	−3.217	**0.001**
NDS	0.009	0.066	−0.122, 0.139	0.131	0.896
Condition_E1 ^b^	0.362	0.311	−0.252, 0.975	1.163	0.245
Condition_E2 ^b^	0.146	0.306	−0.457, 0.750	0.479	0.632
Condition_E3 ^b^	0.137	0.318	−0.489, 0.764	0.433	0.665
Condition_NE ^b^	−0.023	0.315	−0.644, 0.598	−0.073	0.942
Day of week_weekend ^b^	0.091	0.094	−0.094, 0.277	0.971	0.331
Ambient temperature	−0.041	0.014	−0.067, −0.014	−2.965	**0.003**
Condition_E1 × age ^b^	−0.004	0.019	−0.042, 0.034	−0.214	0.830
Condition_E2 × age ^b^	−0.002	0.019	−0.039, 0.035	−0.087	0.931
Condition_E3 × age ^b^	−0.002	0.020	−0.041, 0.036	−0.109	0.913
Condition_NE × age ^b^	0.007	0.019	−0.031, 0.045	0.379	0.705
Condition_E1 × NDS ^b^	0.064	0.086	−0.106, 0.233	0.742	0.458
Condition_E2 × NDS ^b^	0.147	0.085	−0.021, 0.315	0.730	0.084
Condition_E3 × NDS ^b^	0.095	0.088	−0.078, 0.269	1.080	0.280
Condition_NE × NDS ^b^	0.110	0.088	−0.063, 0.283	1.252	0.210
GLMM_2_ (stress-related behavior)					
Intercept ^a^	0.967	0.779	−0.569, 2.503	a	a
Age	−0.075	0.022	−0.119, −0.031	−3.387	**0.001**
NDS	0.037	0.095	−0.151, 0.224	0.386	0.670
Condition_E1 ^b^	−0.901	0.613	−2.109, 0.308	−1.470	0.142
Condition_E2 ^b^	−1.590	0.506	−2.588, −0.592	−3.142	**0.002**
Condition_E3 ^b^	−0.904	0.553	−1.994, 0.187	−1.634	0.102
Condition_NE ^b^	−1.369	0.566	−2.485, −0.253	−2.420	0.016
Day of week_weekend ^b^	−0.568	0.243	−1.048, −0.088	−2.334	**0.020**
Ambient temperature	0.030	0.024	−0.018, 0.077	1.231	0.218
Condition_E1 × age ^b^	0.004	0.037	−0.070, 0.078	0.102	0.919
Condition_E2 × age ^b^	0.072	0.030	0.013, 0.131	2.395	**0.017**
Condition_E3 × age ^b^	0.032	0.034	−0.035, 0.099	0.944	0.345
Condition_NE × age ^b^	0.059	0.034	−0.009, 0.126	1.704	0.088
Condition_E1 × NDS ^b^	0.045	0.163	−0.277, 0.367	0.274	0.784
Condition_E2 × NDS ^b^	0.227	0.143	−0.055, 0.509	1.587	0.113
Condition_E3 × NDS ^b^	0.051	0.151	−0.248, 0.350	0.337	0.736
Condition_NE × NDS ^b^	0.096	0.160	−0.219, 0.411	0.604	0.546
GLMM_4_ (affiliative behavior)					
Intercept ^a^	−1.679	0.882	−3.418, 0.061	a	a
Age	0.002	0.021	−0.039, 0.042	0.089	0.929
NDS	0.358	0.116	0.130, 0.587	3.094	**0.002**
Condition_E1 ^b^	−0.605	0.569	−1.727, 0.517	−1.063	0.288
Condition_E2 ^b^	−1.204	0.659	−2.503, 0.095	−1.827	0.068
Condition_E3 ^b^	−0.493	0.587	−1.651, 0.665	−0.839	0.401
Condition_NE ^b^	0.162	0.615	−1.051, 1.376	0.264	0.792
Day of week_weekend ^b^	−0.075	0.220	−0.508, 0.358	−0.343	0.732
Ambient temperature	0.053	0.027	−0.001, 0.107	1.920	0.055
Condition_E1 × age ^b^	0.007	0.029	−0.050, 0.064	0.237	0.813
Condition_E2 × age ^b^	0.049	0.037	−0.025, 0.123	1.311	0.190
Condition_E3 × age ^b^	0.023	0.032	−0.041, 0.086	0.706	0.480
Condition_NE × age ^b^	−0.032	0.033	−0.098, 0.033	−0.978	0.328
Condition_E1 × NDS ^b^	0.115	0.169	−0.218, 0.449	0.680	0.496
Condition_E2 × NDS ^b^	−0.076	0.209	−0.488, 0.337	−0.362	0.718
Condition_E3 × NDS ^b^	−0.075	0.179	−0.428, 0.278	−0.417	0.677
Condition_NE × NDS ^b^	−0.084	0.178	−0.435, 0.266	−0.474	0.636
GLMM_5_ (agonistic behavior)					
Intercept ^a^	3.712	2.550	−1.327, 8.751	a	a
Age	−0.126	0.041	−0.207, −0.044	−3.052	**0.002**
NDS	−0.260	0.148	−0.552, 0.032	−1.758	0.079
Condition_E1 ^b^	2.317	0.720	0.898, 3.736	3.220	**0.001**
Condition_E2 ^b^	1.759	0.697	0.384, 3.134	2.522	**0.012**
Condition_E3 ^b^	1.666	0.757	0.174, 3.159	2.201	**0.028**
Condition_NE ^b^	1.824	0.727	0.391, 3.257	2.509	**0.012**
Day of week_weekend ^b^	−0.421	0.630	−1.664, 0.822	−0.668	0.504
Ambient temperature	−0.158	0.090	−0.336, 0.020	−1.750	0.080
GLMM_6_ (explorative and scent-marking behavior)					
Intercept ^a^	1.879	0.932	0.040, 3.717	a	a
Age	−0.019	0.026	−0.071, 0.032	−0.739	0.460
NDS	0.316	0.140	0.040, 0.592	2.257	**0.024**
Condition_E1 ^b^	2.003	0.645	0.732, 3.275	3.108	**0.002**
Condition_E2 ^b^	1.339	0.655	0.048, 2.631	2.045	**0.041**
Condition_E3 ^b^	1.467	0.662	0.162, 2.772	2.217	**0.027**
Condition_NE ^b^	1.545	0.719	0.127, 2.964	2.149	**0.032**
Day of week_weekend ^b^	0.102	0.216	−0.325, 0.529	0.471	0.637
Ambient temperature	−0.075	0.029	−0.132, −0.018	−2.607	**0.009**
Condition_E1 × age ^b^	−0.052	0.037	−0.125, 0.022	−1.389	0.165
Condition_E2 × age ^b^	−0.033	0.037	−0.107, 0.041	−0.882	0.378
Condition_E3 × age ^b^	−0.032	0.038	−0.107, 0.043	−0.839	0.402
Condition_NE × age ^b^	−0.051	0.043	−0.135, 0.033	−1.196	0.231
Condition_E1 × NDS ^b^	−0.323	0.179	−0.677, 0.031	−1.802	0.072
Condition_E2 × NDS ^b^	−0.226	0.185	−0.592, 0.139	−1.223	0.221
Condition_E3 × NDS ^b^	−0.274	0.186	−0.642, 0.093	−1.471	0.141
Condition_NE × NDS ^b^	−0.357	0.199	−0.750, 0.035	−1.796	0.073
GLMM_7_ (foraging)					
Intercept ^a^	1.871	0.832	0.230, 3.512	a	a
Age	−0.012	0.025	−0.062, 0.037	−0.493	0.622
NDS	−0.006	0.117	−0.237, 0.224	−0.054	0.957
Condition_E1 ^b^	0.120	0.539	−0.864, 1.264	0.371	0.711
Condition_E2 ^b^	−0.430	0.536	−1.488, 0.627	−0.802	0.422
Condition_E3 ^b^	−0.796	0.636	−2.050, 0.457	−1.253	0.210
Condition_NE ^b^	0.299	0.056	−0.806, 1.404	0.534	0.593
Day of week_weekend ^b^	−0.559	0.250	−1.053, −0.066	−2.235	**0.025**
Ambient temperature	−0.035	0.026	−0.087, 0.016	−1.355	0.176
Condition_E1 × age ^b^	0.016	0.035	−0.054, 0.086	0.442	0.658
Condition_E2 × age ^b^	0.056	0.036	−0.016, 0.127	1.536	0.125
Condition_E3 × age ^b^	0.008	0.039	−0.070, 0.085	0.196	0.845
Condition_NE × age ^b^	0.027	0.038	−0.048, 0.103	0.714	0.475
Condition_E1 × NDS ^b^	−0.075	0.163	−0.396, 0.246	−0.461	0.645
Condition_E2 × NDS ^b^	−0.055	0.169	−0.388, 0.279	−0.323	0.747
Condition_E3 × NDS ^b^	0.155	0.190	−0.219, 0.529	0.816	0.414
Condition_NE × NDS ^b^	−0.206	0.173	−0.546, 0.135	−1.192	0.233

^a^ Not shown as not having a meaningful interpretation. ^b^ These predictors were dummy-coded, with the reference category as follow: Condition: BL; Day of the week: weekdays; Condition × age: BL; Condition × NDS: BL.

**Table 4:** Full results of the Tukey test for the *agonistic* behaviors. Significant results are reported in bold.

Predictors	Estimates	SEM	*ꭓ^2^*	*p*
E1 vs. BL	2.317	0.720	3.220	**0.010**
E2 vs. BL	1.759	0.697	2.522	0.080
E3 vs. BL	1.666	0.757	2.201	0.170
NE vs. BL	1.824	0.727	2.509	0.083
E2 vs. E1	−0.558	0.385	−1.451	0.581
E3 vs. E1	−0.651	0.414	−1.572	0.501
NE vs. E1	−0.493	0.404	−1.222	0.727
E3 vs. E2	−0.093	0.455	−0.204	1.000
NE vs. E2	0.065	0.434	0.149	1.000
NE vs. E3	0.158	0.455	0.346	1.000

## Data Availability

The data presented in this study is available on request from the corresponding author.
